# Recognition of Anesthetic Barbiturates by a Protein Binding Site: A High Resolution Structural Analysis

**DOI:** 10.1371/journal.pone.0032070

**Published:** 2012-02-16

**Authors:** Simon Oakley, L. Sangeetha Vedula, Weiming Bu, Qing Cheng Meng, Jin Xi, Renyu Liu, Roderic G. Eckenhoff, Patrick J. Loll

**Affiliations:** 1 Department of Anesthesiology and Critical Care, University of Pennsylvania, Philadelphia, Pennsylvania, United States of America; 2 Department of Biochemistry and Molecular Biology, Drexel University College of Medicine, Philadelphia, Pennsylvania, United States of America; International Centre for Genetic Engineering and Biotechnology, Italy

## Abstract

Barbiturates potentiate GABA actions at the GABA_A_ receptor and act as central nervous system depressants that can induce effects ranging from sedation to general anesthesia. No structural information has been available about how barbiturates are recognized by their protein targets. For this reason, we tested whether these drugs were able to bind specifically to horse spleen apoferritin, a model protein that has previously been shown to bind many anesthetic agents with affinities that are closely correlated with anesthetic potency. Thiopental, pentobarbital, and phenobarbital were all found to bind to apoferritin with affinities ranging from 10–500 µM, approximately matching the concentrations required to produce anesthetic and GABAergic responses. X-ray crystal structures were determined for the complexes of apoferritin with thiopental and pentobarbital at resolutions of 1.9 and 2.0 Å, respectively. These structures reveal that the barbiturates bind to a cavity in the apoferritin shell that also binds haloalkanes, halogenated ethers, and propofol. Unlike these other general anesthetics, however, which rely entirely upon van der Waals interactions and the hydrophobic effect for recognition, the barbiturates are recognized in the apoferritin site using a mixture of both polar and nonpolar interactions. These results suggest that any protein binding site that is able to recognize and respond to the chemically and structurally diverse set of compounds used as general anesthetics is likely to include a versatile mixture of both polar and hydrophobic elements.

## Introduction

General anesthetics, either inhaled or injected, are mainstays of modern surgery. They are widely used to reversibly decrease patients' sensation of otherwise painful and unpleasant medical procedures. General anesthetics function by decreasing synaptic transmission of action potentials, primarily within the central nervous system. Overdoses of anesthetics, though rare in clinical practice, are potentially lethal as these drugs progressively recruit other “off-pathway” actions, such as cardiovascular and respiratory depression.

The original anesthetic agents were discovered serendipitously and then served as lead compounds for a decades-long process that has resulted in the more efficacious and somewhat safer agents that are used today. This progress occurred without definitive knowledge of the pharmacological targets of these drugs; indeed, even today questions remain as to which targets are the most clinically relevant. However, it is widely believed that general anesthetics act at multiple protein sites [1] and that these sites include membrane-bound channel proteins within the central nervous system. Likely candidates include the Cys-loop ligand-gated ion channels [2], [3], some potassium channels [4], and the mitochondrial respiratory chain [5].

Many of these potential anesthetic targets are predicted to be dominated by alpha helical structure, particularly in their membrane-spanning regions. These regions typically contain bundles of helices, associating side-to-side in a parallel or antiparallel manner. In the rare cases where genetic or biochemical mapping data are available, the anesthetic-sensitive residues have been found to be located in these membrane-spanning regions, in areas predicted to lie between adjacent subunits [6], [7], [8], [9], [10], [11], [12], [13]. However, the details of these binding sites, including how they recognize anesthetics, will not be fully appreciated until high-resolution structures are obtained for pharmacologically relevant targets. Unfortunately, this goal may not be realized for some time, owing to the difficulties associated with expressing, purifying, and crystallizing large eukaryotic membrane proteins [14].

Until structures become available for mammalian membrane-bound anesthetic targets, it can be fruitful to use surrogate proteins as models for protein-anesthetic recognition. Several soluble proteins of known structure contain binding sites that recognize a wide variety of anesthetic agents, including serum albumin [15], firefly luciferase [16], and synthetic peptides [17]. In addition, GLIC, a prokaryotic homolog of eukaryotic ligand-gate ion channels, has been shown to be inhibited by various anesthetics [18], and its structure has recently been determined in complex with two such drugs [19]. However, an arguably more useful surrogate is horse-spleen apoferritin, because it binds both inhaled and injected general anesthetics with affinities that are clinically relevant, and which correlate well with clinical measures of anesthetic potency [20], [21]. Further, apoferritin is commercially available, very stable, and crystallizes readily, making for a tractable model system.

We have termed apoferritin a “unitary” binder of anesthetics because its single binding site is able to recognize anesthetic compounds belonging to widely varying chemotypes [21]. These compounds are bound with affinities that correlate well with their potencies, arguing that the apoferritin site shares physicochemical features with the binding sites in clinically relevant targets. Furthermore, in a homologous series of analogs of the injectable anesthetic propofol, each compound's affinity for apoferritin was found to be strongly correlated with its ability to potentiate GABA responses at the GABA_A_ receptor [22], suggesting that the apoferritin anesthetic binding site may specifically resemble that of the GABA_A_ receptor. Selectivity for this chemiarchitecture is demonstrated by the fact that non-immobilizers do not bind the apoferritin site [23]. In addition to these pharmacological similarities, the apoferritin site may share topological elements in common with anesthetic binding sites in human membrane-bound ion channels, since the apoferritin site lies at the interface between two helical bundle domains.

**Figure 1:**
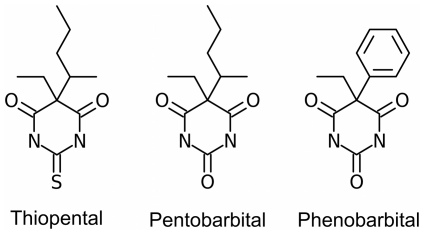
Chemical structures of selected barbiturates.

Barbiturates were discovered during the first decades of the twentieth century, and rapidly became widely used as sedatives and general anesthetics [24]. Since reaching their heyday in the 1930’s and 1940’s, they have been largely replaced by propofol and other sedatives, such as the benzodiazepines, which are less prone to overdose, abuse, misuse and addiction. However, phenobarbital is still used as a sleep aid, thiopental is still used as an induction agent, and pentobarbital is still widely used in veterinary practice (see Figure 1 for the chemical structures of these compounds). The molecular details of how barbiturates interact with their targets remain largely unknown, as there exist no structures for protein-barbiturate complexes in the Protein Data Bank. Here, we show that pentobarbital and thiopental, like several other general anesthetics, bind to a common cavity in apoferritin. This result further supports the notion that apoferritin is a unitary recognizer of GABAergic anesthetics, and validates the protein's use as a surrogate model for the clinically relevant binding sites of general anesthetics.

## Results

### Apoferritin binds barbiturates

Isothermal titration calorimetry (ITC) was used to confirm that phenobarbital, pentobarbital, and thiopental bind directly to apoferritin, with dissociation constant values lying in the range 10 to 700 µM (Table 1). Thiopental binds most tightly; the affinity of pentobarbital is approximately 6-fold lower, while phenobarbital's affinity for apoferritin is 50-fold lower than that of thiopental. All compounds display favorable enthalpies of binding. The affinities of the barbiturates for apoferritin are very similar to reported EC_50_ values for general anesthesia in mammals and for enhancement of GABA_A_ activity (Table 1).

**Table 1:** Binding, physical, and activity data for barbiturates.

	Thiopental	Pentobarbital	Phenobarbital
Dissociation constant *K*_d_ for apoferritin binding (µM)	11.0±0.9	64.3±5.5	672±27
Enthalpy of binding to apoferritin (kcal/mol)^a^	−7.3±0.4	−4.4±0.7	—
apoferritin cavity volume (Å^3^)	388.6	407.6	—
Ligand volume (Å^3^)	223.9	210.5	211.3
Packing density	0.58	0.52	—
EC_50_ for anesthesia (µM)	25^b^	50^b^	590^c^
EC_50_ for GABA potentiation (µM)	20^d^	30–60^d^^, ^ e ^, ^ f	300–900^e^^, ^ f
logP^g^	2.9	2.1	1.5

*^a^*Because of the low affinity of phenobarbital for apoferritin, it was not possible to obtain a satisfactory estimate of the molar binding enthalpy.

*^b^* [16].

*^c^* [41].

*^d^* [42].

*^e^* [43].

*^f^* [44].

*^g^*Calculated using XLOGP3 [45].

The methylbutyl chain found in pentobarbital and thiopental can exist as one of two optical isomers, with the stereocenter at the branch point between the methyl and butyl groups; the difference in anesthetic potency between the two stereoisomers is small (roughly 2-fold), with the *S*-enantiomer being the more potent [25]. Racemic mixtures of these compounds are used clinically. To probe the effect of barbiturate stereochemistry on apoferritin binding, we purified the optical isomers of thiopental and measured their binding affinities using ITC (Table S1). The *S*-enantiomer was found to bind 1.5-fold more tightly than the *R*-form, mirroring the clinically observed difference in potencies.

### Structure determination

To determine the site at which the barbiturates bind, apoferritin was co-crystallized with thiopental and pentobarbital and the crystal structures of the complexes were determined (Table 2; because of phenobarbital's low affinity for apoferritin, the crystal structure of this complex was not attempted). The crystals that we obtained had space groups and unit cells isomorphous with previous apoferritin structures, and structure determination via molecular replacement confirmed the same crystal system. Initial electron density maps contained clear density in the anesthetic binding site, confirming the presence of the barbiturate ligands. For both pentobarbital and thiopental, the initial electron density of the ligand was distinct from that seen in the unliganded structure, where the binding site contains four water molecules [21]. In both complexes, the electron density corresponding to the barbiturate ring was more distinct than the density for the alkyl substituents, suggesting that the ring structure may be held more tightly than the ethyl and methylbutyl chains.

**Table 2:** Crystallographic data collection and refinement statistics.

	Pentobarbital	Thiopental
***Data collection***		
Spacegroup	*F*432	*F*432
Wavelength (Å)	0.977	1.542
Resolution range^a^	45.62-1.90(1.97-1.90)	41.79-2.00(2.05-2.00)
Cell constants *a = b = c* (Å)	182.48	182.18
Number of unique reflections	21,095	33,018
Mean redundancy	34.09 (34.09)	21.2 (8.5)
Completeness (%)	100.0 (100.0)	99.7 (98.4)
*R*_MERGE_	0.109 (0.527)	0.061 (0.616)
Mean *I*/ó(*I*)	20.0 (7.8)	40.6 (3.8)
***Refinement***		
*R*_WORK_	0.174	0.172
*R*_FREE_	0.208	0.193
Number of atoms		
total	1575	1539
protein	1346	1344
ligand	16	16
water	197	164
metal	6	5
other solvent atoms	10	10
RMS deviation from ideality		
bonds (Å)	0.007	0.007
angles (deg)	0.959	0.936

*^a^*Values in parentheses correspond to the outermost resolution shell.

Apoferritin forms a highly symmetric 24-mer. Each protomer in the oligomer forms a helical bundle composed of four long antiparallel helices, with a fifth helix capping one end of the bundle, and an extended crossover strand connecting the second and third helices. Although our apoferritin preparation contains both H- and L-chains, only the L-protomers have been modeled in this structure (see Methods). The overall structure of the apoferritin molecule is not significantly altered in response to barbiturate binding; root-mean-square differences in Cα positions are less than 0.1 Å when either barbiturate complex structure is superimposed upon the unliganded apoferritin structure.

The apoferritin anesthetic binding site is located within a cavity at the interface between two protomers; the cavity is largely buried within the protein interior, but is connected to the protein surface (and thus to bulk solvent) via two small funnel-shaped openings. The two protomers forming the cavity are related by a 2-fold symmetry axis that runs through the center of the binding site. Hence, equivalent residues from both subunits contribute to the cavity surface, and an anesthetic molecule bound within this cavity has the potential to interact with both copies of a given residue. The symmetry of the binding site dictates that the ligand can occupy two positions, related to one another by a 180° rotation. These two positions overlap, so that in a given cavity the ligand can adopt only one of the two symmetry-related conformations; however, because the electron density is averaged over the entire crystal, the map will reflect both conformations.

### Thiopental binding site

**Figure 2:**
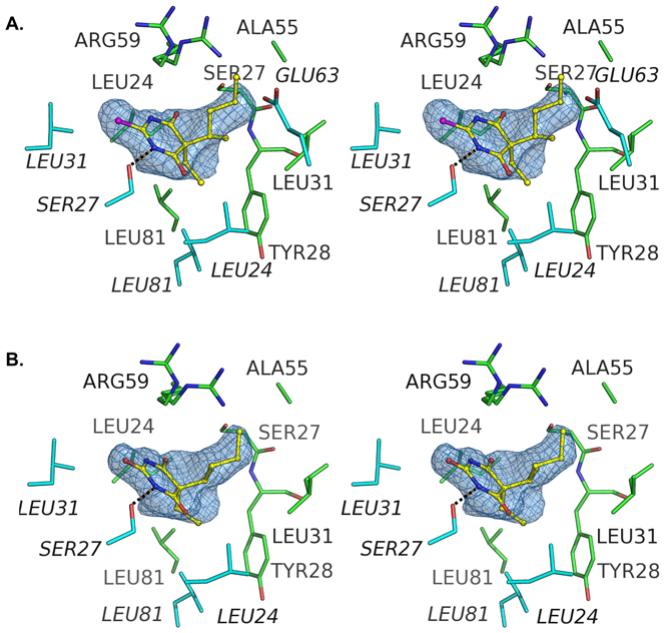
Stereoviews showing the anesthetic binding site of apoferritin with A) thiopental and B) pentobarbital. The hydrogen bond between the drug and Ser-27 is shown as a black dashed line. In both images the ligands are depicted as sticks; color code: carbon, yellow; nitrogen, blue; oxygen, red; and sulfur, magenta. The electron density shown is 2*F*o-*F*c density calculated from the final refined structures, contoured at sigma level of 0.8 and carved around the ligands. Figures 2 and 3 were generated using PyMOL (The PyMOL Molecular Graphics System, Schrödinger, LLC).

Thiopental interacts with apoferritin via a hydrogen bond, a possible electrostatic interaction, and multiple hydrophobic contacts. The hydrogen bond is formed between the ring nitrogen of the barbiturate and Ser-27 (Figure 2A). The geometry of this hydrogen bond is close to ideal; the oxygen-nitrogen distance is 2.87 Å and the serine's hydroxyl oxygen lies close to the plane of the barbiturate ring (0.002 Å perpendicular distance from the oxygen to the ring plane). The angle formed by the barbiturate ring nitrogen, the serine hydroxyl oxygen, and the serine beta carbon is 111.9°, also highly favorable [26].

An electrostatic interaction may occur between the barbiturate ring sulfur and the guanidino group of Arg-59. The Arg-59 side chain is present in two conformations: one similar to the “closed” conformation seen in the water-bound (unliganded) apoferritin structure, the other to the “open” conformation found in the apoferritin-propofol complex structure [21]. In the pentobarbital complex, the closed conformer of the arginine places a guanidino nitrogen 3.8 Å from the barbiturate ring sulfur. No electron density is observed for any other nearby counter-ion. This sulfur atom is capable of a keto-enol tautomeric transition, and is assigned a formal charge of -1 in the enol form. We cannot directly determine whether the drug adopts the keto or enol form in our structure, because the structural differences between the two forms are minute. Although the close presence of the Arg-59 guanidino group suggests that the sulfur could possess at least partially negative character, the generally hydrophobic nature of the ligand binding pocket makes it likely that the ligand adopts the less polar keto form.

Hydrophobic interactions occur between the protein and the ethyl and methylbutyl chains that extend from the thiopental ring. The ethyl group of the thiopental ligand sits atop a hydrophobic patch within the binding site that is created by the side chains of Tyr-28 and Leu-81 from one apoferritin monomer and Leu-24 and Leu-81 from the symmetry-related apoferritin molecule. The atoms in the ligand and protein that participate in this interaction are all nonpolar and the distances between interacting atoms range from 3.7 to 4.5 Å. The closest polar group to ligand's ethyl chain is the carbonyl group of Leu-24, which is 5.0 Å from the thiopental.

The methylbutyl group of the ligand lies in the center of the hydrophobic cavity, with its tail extending into the funnel-shaped aperture that connects the anesthetic binding site with the surface of the protein. It packs against methylene groups of the Arg-59 side chain; this arginine side chain forms much of the cap that closes off the anesthetic binding site from solvent. Both stereoisomers were consistent with the electron density in the binding site; given their similar affinities, the ligand was modeled as a mixture of *R*- and *S*-forms of the molecule. The majority of the methylbutyl group lies in a position that overlaps electron density corresponding to the barbiturate ring of the symmetry-related copy of the ligand. This fact, together with the presumed presence of different optical isomers, means that the precise positions of the atoms in the methylbutyl group are not well determined.

### Pentobarbital binding

Pentobarbital binds apoferritin in a manner that is directly equivalent to that seen with thiopental. A similar hydrogen bond is seen between a barbiturate ring nitrogen and the hydroxyl moiety of Ser-27 (nitrogen-oxygen distance = 2.83 Å; distance between the serine hydroxyl oxygen and the plane of the barbiturate ring = 0.4 Å; angle formed by Cβ27—Oγ27—barbiturate ring nitrogen = 111.2°; Figure 2B). As is the case with thiopental, Arg-59 adopts two alternate conformers in the pentobarbital complex structure, and a guanidino nitrogen of one conformer lies 3.6 Å from the ring oxygen, contributing a possible electrostatic component to pentobarbital recognition. The ethyl and methylbutyl chains of the pentobarbital molecule lie in similar positions to those occupied by their counterparts in the thiopental structure, and make similar interactions with the protein. An apparent steric clash exists between the methylbutyl group and the hydroxyl group of Ser-27 (carbon-oxygen distance 2.6 Å). This may merely reflect a lack of precision in positioning the atoms in the methylbutyl group, similar to that encountered with thiopental; alternatively, this copy of Ser-27 (the symmetry mate of the Ser-27 side chain that is hydrogen bonding to the barbiturate ring) may adopt an alternate conformer that we were unable to discern in the electron density.

## Discussion

A consensus now exists that general anesthetics exert their effects through binding and modulating the activities of ion channels and other integral membrane proteins. Unfortunately, despite recent successes [19], it remains difficult to visualize the details of how anesthetics interact with these targets, since integral membrane proteins are challenging systems for high resolution structural studies. Until structure determination for membrane proteins becomes routine, surrogate model systems will remain valuable tools for advancing our understanding of how proteins recognize anesthetics. Apoferritin arguably represents one of the most useful such models now available. Its structure is well characterized, and it recognizes a wide variety of inhaled and intravenous anesthetic agents. Importantly, apoferritin's binding affinities for different anesthetics are comparable to clinically relevant concentrations; in contrast, the prokaryotic ion channel GLIC displays very different patterns of anesthetic recognition than mammalian anesthetic targets [18], despite possessing a pentameric architecture that is similar to those of known mammalian targets such as the acetylcholine and GABA_A_ receptors [27].

Barbiturates can now be added to the list of general anesthetics recognized by apoferritin, providing a second example of recognition of an *injected* general anesthetic (the first being propofol). Interestingly, both propofol and the barbiturates act largely via GABA transmission, as both can directly gate and potentiate the inhibitory GABA_A_ receptor. For propofol and related compounds, affinity for apoferritin is very close to the EC_50_ for GABA potentiation [21]; the same appears true for barbiturates. EC_50_ values for GABA potentiation by thiopental, pentobarbital, and phenobarbital are close to their *K*_d_ values for apoferritin (Table 1), bolstering the argument that the anesthetic binding sites on apoferritin and the GABA_A_ receptor share structural and physicochemical similarity.

Within the barbiturate class, different compounds potentiate GABA effects and mediate anesthesia with different potencies; these differences are largely mirrored in their affinities for apoferritin. It is therefore interesting to ask which features of the apoferritin-barbiturate interaction are responsible for these differences in affinity. Thiopental and pentobarbital are iso-structural, differing only at a single atom, and adopt essentially identical orientations and conformations within the apoferritin binding site. The slightly larger sulfur atom gives thiopental a slightly larger volume and surface area, but such trivial size differences seem unlikely to explain an approximate five-fold difference in affinity. A more likely explanation is thiopental's more hydrophobic nature, as reflected by its higher partition coefficient (Table 1). If indeed the apoferritin binding site preferentially recognizes thiopental on the basis of its lower polarity, then this argues that the form of the anesthetic bound to the protein is the less polar keto tautomer.

Phenobarbital has a substantially lower affinity for apoferritin than either thiopental or pentobarbital. While it is similar to in size to the other two compounds, it is more polar than either, consistent with an apoferritin binding site that prefers less polar ligands. Also, modeling suggests that phenobarbital's rigid phenyl ring could lead to steric clashes between the ligand and the protein; the methylbutyl substituent of thiopental and pentobarbital, while not much smaller than the phenyl ring, is substantially more flexible, and hence more easily accommodated within the binding site.

**Figure 3:**
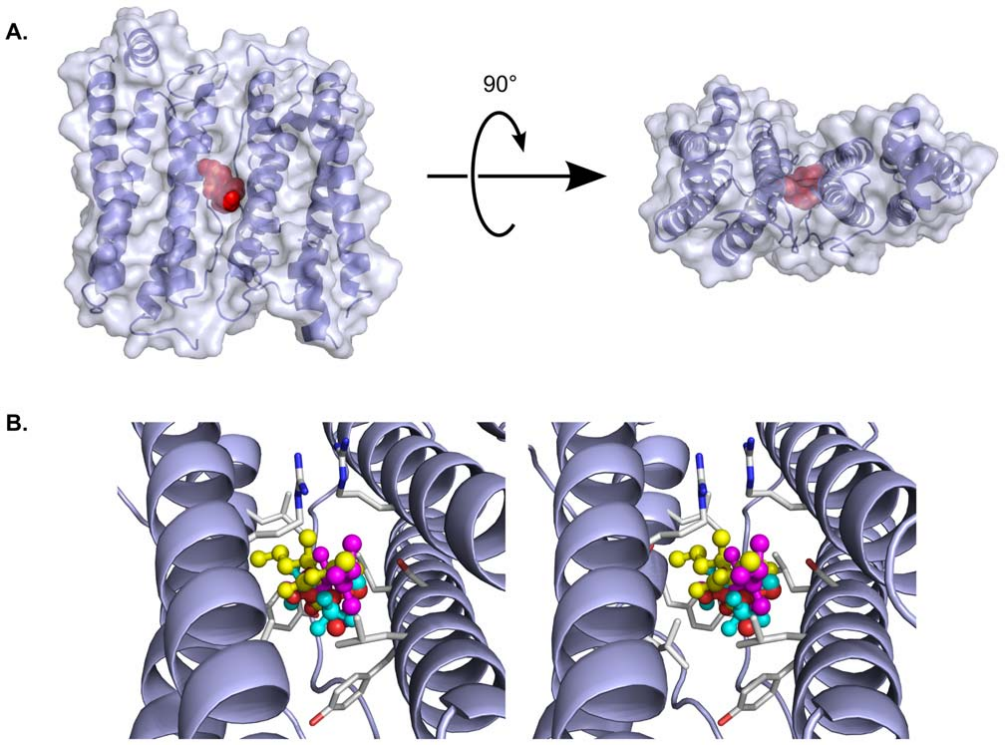
Diverse general anesthetics utilize a common binding site in apoferritin. A) Orthogonal views of the apoferritin dimer, shown as a partially transparent molecular surface encasing a cartoon representation of the backbone. The anesthetic binding site is marked by thiopental, which is shown in a red space-filling representation. B) Stereo view showing a close-up of the anesthetic binding site, in an orientation similar to that seen in the left half of the upper panel. Four different general anesthetics are shown in ball-and-stick representations: Thiopental (yellow); propofol (magenta); isoflurane (cyan); and halothane (orange). The protein backbone is shown in blue, while selected protein side chains are shown in light gray. All four compounds, despite belonging to different chemotypes, utilize the same binding cavity, in which their positions overlap extensively.

There are both similarities and differences in how apoferritin recognizes barbiturates versus other, chemically distinct classes of anesthetics (Figures S3 and S4). Similarity can be found in a common binding area shared by haloalkanes, halogenated ethers, propofol analogs, and barbiturates; this area is a small hydrophobic patch created by Tyr-28, Leu-24, and Leu-81 (Figures 3, S3, and S4). In the barbiturate complexes this hydrophobic patch interacts with the ethyl substituent, in the propofol complexes this position is occupied by an aromatic ring, and in the halothane and isoflurane complexes it is occupied by two fluorine atoms (Text S1).

Differences in ligand recognition exist between the apoferritin-barbiturate and apoferritin-propofol complex structures. Even though both propofol and the barbiturates contain aromatic rings, the planes of these rings are rotated nearly 90 degrees between the two classes of drug. The highly hydrophobic propofol analogs bind with their hydroxyl groups (their only potential hydrogen bond donor/acceptor) pointing toward the opening of the protein cavity, away from the cavity walls, and do not use this hydroxyl group to interact with the protein. In contrast, the position of the barbiturates allows them to hydrogen bond with Ser-27 of apoferritin. This hydrogen bond, together with a potential polar interaction between the barbiturate ring and Arg-59, are the most notable differences between how apoferritin recognizes the barbiturates and how it recognizes other general anesthetics. Apoferritin appears to rely principally upon van der Waals interactions when binding other anesthetics, with little or no contribution from polar interactions. van der Waals interactions do contribute to recognition of the barbiturates, particularly between the alkyl substituents of the drugs and hydrophobic side chains of the protein. However, the favorable enthalpies of binding provided by the polar interactions are likely to substantially exceed the contribution from these van der Waals interactions, and dominate the binding energies.

The exploitation of polar interactions for barbiturate recognition has interesting implications for anesthetic binding. First, it means that the apoferritin site (and probably any site that responds to multiple classes of general anesthetics) must be versatile, and able to rely to differing degrees upon hydrophobic versus polar interactions, depending upon the ligand. Such an amphipathic site should be able to recognize different targets in a versatile manner [28]. For example, apoferritin contains polar side chains within its hydrophobic cavity, but these side chains are capable of interacting with partners other than the ligand. In the absence of ligand, the hydroxyl group of Ser-27 can hydrogen bond with a backbone carbonyl oxygen of the protein; in the presence of barbiturates, this same side chain interacts with the drug. In this way, anesthetics can competitively alter hydrogen-bonding patterns within their binding site, and potentially affect protein stability or dynamics [29].

Another way in which proteins can flexibly recognize different ligands is by stretching or shrinking their binding pockets. However, proteins are limited in the degree to which they can expand or contract a cavity before their structural integrity is compromised. Thus, the volume of the anesthetic-binding cavity in apoferritin varies to some degree when binding different ligands, but affinity is reduced for ligands that are too large to be comfortably accommodated within the acceptable range of cavity volumes. Interestingly, thiopental and pentobarbital are larger than other apoferritin anesthetic ligands examined to date, and yet they are still bound with reasonably high affinity. We suggest that an explanation lies with the polar interactions that the barbiturates make within the apoferritin binding pocket.

**Figure 4:**
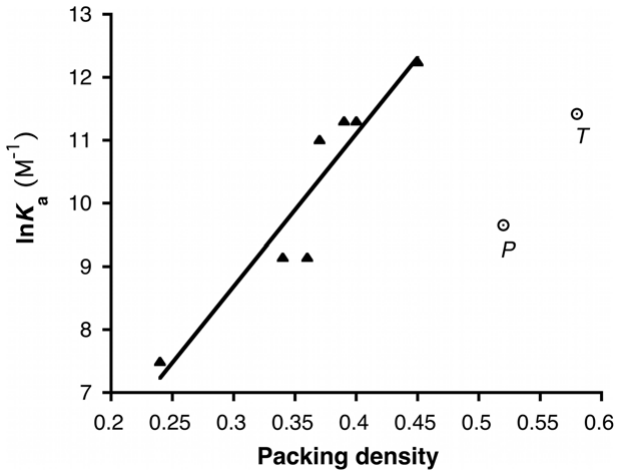
The packing density/affinity relationship for barbiturates is offset from that of the propofol-like compounds, interpreted here as the addition of an entropic penalty offset by new enthalpic gains. Shown is the relationship between packing volume (fraction of cavity volume occupied by ligand) and affinity (dissociation constant, *K*_d_) for apoferritin. The filled triangles represent a series of propofol analogs, for which a linear dependence of affinity upon packing density has been documented [21]. The open circles represent the barbiturates (*P*, pentobarbital; *T*, thiopental).

For ligands that rely solely on van der Waals interactions for recognition, larger ligands have larger surface areas, and can therefore develop larger favorable enthalpies of binding; however, they will also be bound more snugly in the binding pocket, imposing an entropic penalty. At some point, the incremental enthalpic gains associated with increased surface area will be outweighed by the increasing entropic penalty. For nonpolar ligands, the competing enthalpic and entropic effects lead to maximum affinity when the ligand's volume corresponds to approximately 50–60% of the available cavity volume [30]. In contrast, strong polar interactions between a ligand and its target contribute substantially larger favorable enthalpies of binding than do van der Waals interactions, and so when polar interactions are present, greater losses in conformational entropy (and hence higher packing densities) can be tolerated. This appears to be the case with anesthetic binding to apoferritin (Figure 4). For propofol-like compounds that make no polar interactions with the protein, affinity decreases when ligand volume is increased beyond 50% of the cavity volume [21]. However, for compounds like thiopental and pentobarbital that make polar contacts with apoferritin, ligands can fill well over 50% of the available cavity volume, yet still bind with high affinity. Unfortunately, it is not possible to extract the loss in ligand conformational entropy from our calorimetric data, since calorimetric estimates of ΔS include contributions from the displacement of water.

We caution that understanding why a particular anesthetic binds with high affinity to a given target will not necessarily provide insights into general anesthesia, since anesthetic side effects are presumably also mediated by interactions with protein targets, some of which may occur with high affinity. However, the side-effect profiles of currently-used anesthetics vary considerably, suggesting that, unlike anesthetics' clinically useful effects, side effects tend to derive from chemotype-specific interactions. Therefore, targets that recognize many different anesthetic chemotypes are likely to reveal binding properties that are relevant to the production of the anesthetized state.

In summary, we present the first structures of barbiturates bound to a specific protein target. They add support to the notion that direct protein interactions subserve anesthetic effects. Further, these data emphasize that apoferritin is a unitary anesthetic binder, capable of accommodating different general anesthetic chemotypes. Its binding site contains a three-dimensional distribution of amphipathic features that allow recognition of a diverse collection of anesthetic ligands with remarkably high, and clinically relevant, affinity. Thus, it would seem that the apoferritin site can be considered a prototypical anesthetic binding site, and used for purposes as diverse as database mining or screening of novel anesthetic candidates.

## Materials and Methods

### Materials

Apoferritin, thiopental, pentobarbital, and phenobarbital were purchased from Sigma-Aldrich (St. Louis, MO, USA). Optical isomers of thiopental were isolated using a Nucleodex Beta-PM chiral column (10×250 mm; 5 µm pore size), using a mobile phase of 40% acetonitrile in 1.0% triethylammonium acetate, pH 4 and a flow rate of 0.6 mL/min.

### Isothermal titration calorimetry

ITC measurements were carried out essentially as previously described [20], using a MicroCal VP-ITC instrument (Northampton, MA). The sample cell (1.43 mL) contained 10 µM apoferritin 24-mer that had been dialyzed overnight against 20 mM NaHPO_4_ pH 7.0 containing 130 mM NaCl. The reference cell contained water. Ligands were dissolved in the dialysate, to a final concentration of 0.45–1.6 mM, and filtered through 0.2 µm PTFE syringe filters. The titration volumes were 12 µL for the first injection, followed by 14 µL each for the subsequent injections. Sequential titrations were performed until the heat change was essentially constant. Sequential titrations were linked using ConCat32 software (Microcal, Inc.), and corrections were applied for heat changes due to buffer-buffer, buffer-protein, and ligand-buffer dilutions. Representative ITC traces are shown in Figure S5.

### Protein crystallization and data collection

Apoferritin was purified by gel filtration chromatography with 0.2 M NaOAc, pH 5.0 as the mobile phase, and concentrated to a final concentration of 12 mg/mL for co-crystallization with thiopental. For co-crystallization with pentobarbital, apoferritin from Sigma was diluted with deionized water to a final concentration of 12 mg/mL. 1–2 µL of reservoir solution containing 0.2–1.6 M (NH_4_)_2_SO_4_ and 0.1–0.275 M CdSO_4_ were mixed with equal volumes of apoferritin solution and equilibrated over 0.7–1 mL reservoir solution at 18°C. Thiopental (0.5 mM) was dissolved directly in the reservoir solutions prior to crystallization trials, while pentobarbital (1–3 mM) was added to the protein solution and incubated for 30 min prior to crystallization trials. All solutions were pre-filtered through a 0.2 µm filter. Crystals appeared within 1–2 weeks and grew to a final size of 200–300 µm. Single crystals with well-defined edges were cryoprotected using reservoir solution containing 0.5–3 mM ligand and 30% glycerol and flash-cooled in liquid nitrogen for data collection. Diffraction data were collected at beamline X6A of the National Synchrotron Light Source, and integrated using d*TREK [31].

### Structure determination

Molecular replacement was carried using a poly-alanine search molecule derived from the unliganded apoferritin structure, with all water molecules and metal ions removed; both the thiopental and pentobarbital complex structures were determined using the PHASER molecular replacement program [32]. Initial 2*F_O_-F_C_* and *F_O_-F_C_* electron density maps showed features corresponding to side chains, water molecules, and metal ions, as well as the barbiturate compounds bound in the anesthetic binding site. The structures were refined by iteratively cycling between manual rebuilding and automated refinement. Atomic coordinates of side chains, water molecules and cadmium ions were built into 2*F_O_-F_C_* electron density maps using COOT [33]. The atomic positions, temperature factors and occupancies were refined using PHENIX [34] and REFMAC [35]. Towards the end of the refinement the barbiturate compounds were built into electron density at the anesthetic binding site. The electron density for both structures indicated the presence of two optical isomers, which were incorporated into the models. The quality of phase information was assessed by generating omit maps with PHENIX and SFCHECK [36], and stereochemistry was validated using PROCHECK [37] and the validation tools within COOT. Commercially available apoferritin is a mixture of heavy and light chains, in an approximate mole ratio of 1∶6; only the light chain can be observed in the electron density map, as has previously been demonstrated [38]. Coordinates and structure factors have been deposited with the PDB (thiopental, PDB ID 3RD0; pentobarbital, PDB ID 3RAV).

### Structure analysis

Anesthetic binding pocket volumes were calculated as described ([21]; Text S1; Figures S1 and S2) using VOIDOO [39] and standard values for atomic radii [40]. Ligand volumes were obtained by minimizing ligand structures with PHENIX [34], after which the volume of the energy-minimized ligand structure was calculated using VOIDOO.

## Supporting Information

Figure S1 **Calculated cavity volume versus VOIDOO iteration, for two apoferritin structures.** In early cycles, where the grid used to calculate volume is coarse, the determined cavity volumes are underestimated. With increasing iterations the grid becomes finer and detected cavity volume increases and eventually converges. The small diamonds represent ten random orientations of the unliganded apoferritin structure (PDB ID 3F32); the crosses represent ten random orientations of the pentobarbital structure. The curves represent gnuplot fits of equation (1) to the data (see Text S1). The arrows indicate periods of local decline, which may cause the VOIDOO program to terminate prematurely.
(TIF)

Figure S2 **Comparison of two methods for determining apoferritin cavity volume.** The crosses represent volumes calculated by choosing ten random orientations of the molecule, allowing VOIDOO to run until program termination, and averaging the volumes generated for each orientation. The squares represent volumes calculated from *v(n)* curves fitted to the VOIDOO output, using *n* = 25 (see Text S1). The overall trend is maintained for the two methods, but volumes calculated by averaging cavity volumes reported after program termination were in all cases smaller than volumes calculated by curve fitting. PDB ID codes of apoferritin-anesthetic complexes contributing to this figure: 3F32, unliganded; 1XZ1, halothane; 1XZ3, isoflurane; 3F33, propofol; 3RD0, thiopental; 3RAV, pentobarbital; 3F34 & 3F35, 2,6-diethyl phenol; 3F36, 2-isopropyl phenol; 3F37 & 3F38, 2,6-dimethyl phenol; 3F39, phenol.
(TIF)

Figure S3 **Stereo diagrams showing the hydrophobic anesthetic binding cavity of apoferritin bound to diverse general anesthetics.** Pentobarbital is shown in both images, to allow direct comparison. The molecular surface of the cavity is shown as a semi-transparent tan surface that partially obscures Leu-24. A) The pentobarbital-apoferritin complex; both optical isomers are shown in this panel, in yellow and cyan. (In subsequent panels, only the more abundant isomer of pentobarbital is shown, for the sake of clarity). B) An overlay of propofol (yellow, PDB entry 3F33) and pentobarbital (cyan).
(TIF)

Figure S4 **Stereo diagrams showing the hydrophobic anesthetic binding cavity of apoferritin bound to diverse general anesthetics (continued from Figure S3).** Pentobarbital is included in both images, to allow direct comparison. The molecular surface of the cavity is shown as a semi-transparent tan surface that partially obscures Leu-24. A) Overlay of halothane (yellow) and pentobarbital (cyan). B) Overlay of isoflurane (yellow) and pentobarbital (cyan).
(TIF)

Figure S5 **Representative ITC traces for the binding of barbiturates to apoferritin.** Panel A, thiopental; panel B, pentobarbital; panel C, phenobarbital.
(TIF)

Table S1 Apoferritin binding affinities of the optical isomers of thiopental.
(DOC)

Text S1 **Details of cavity volume calculations, and a comparison of how different general anesthetics to the common apoferritin pharmacophore.**
(DOC)
