# Supplementary figures and images for: Recognition of Anesthetic Barbiturates by a Protein Binding Site: A High Resolution Structural Analysis
Source: PLoS One. 2012 Feb 16;7(2):e32070. doi: 10.1371/journal.pone.0032070 (PMC3281113; doi:10.1371/journal.pone.0032070)

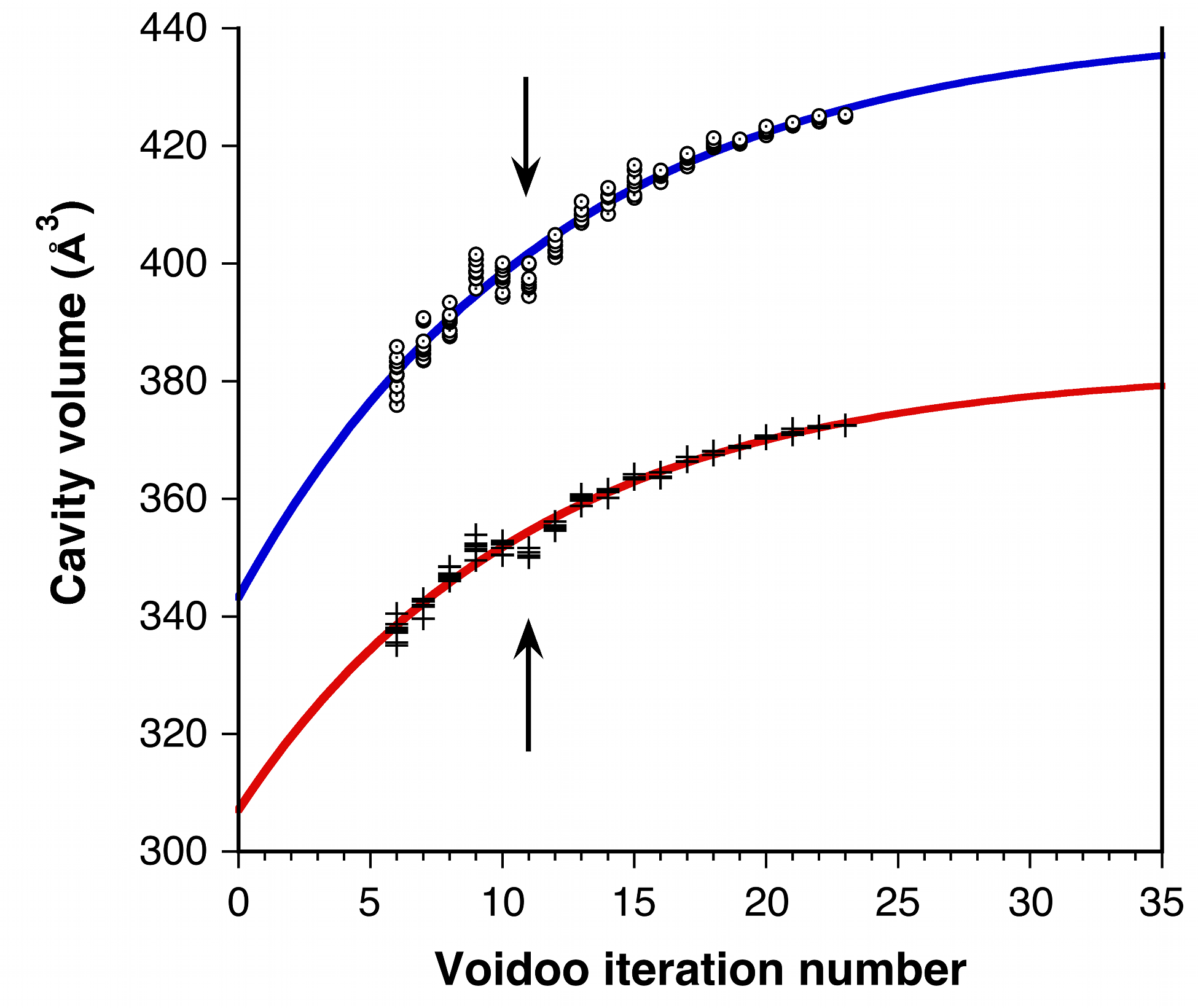

Supplement: Figure S1 — Calculated cavity volume versus VOIDOO iteration, for two apoferritin structures. In early cycles, where the grid used to calculate volume is coarse, the determined cavity volumes are underestimated. With increasing iterations the grid becomes finer and detected cavity volume increases and eventually converges. The small diamonds represent ten random orientations of the unliganded apoferritin structure (PDB ID 3F32); the crosses represent ten random orientations of the pentobarbital structure. The curves represent gnuplot fits of equation (1) to the data (see Text S1). The arrows indicate periods of local decline, which may cause the VOIDOO program to terminate prematurely. (TIF) [file pone.0032070.s001.tif]

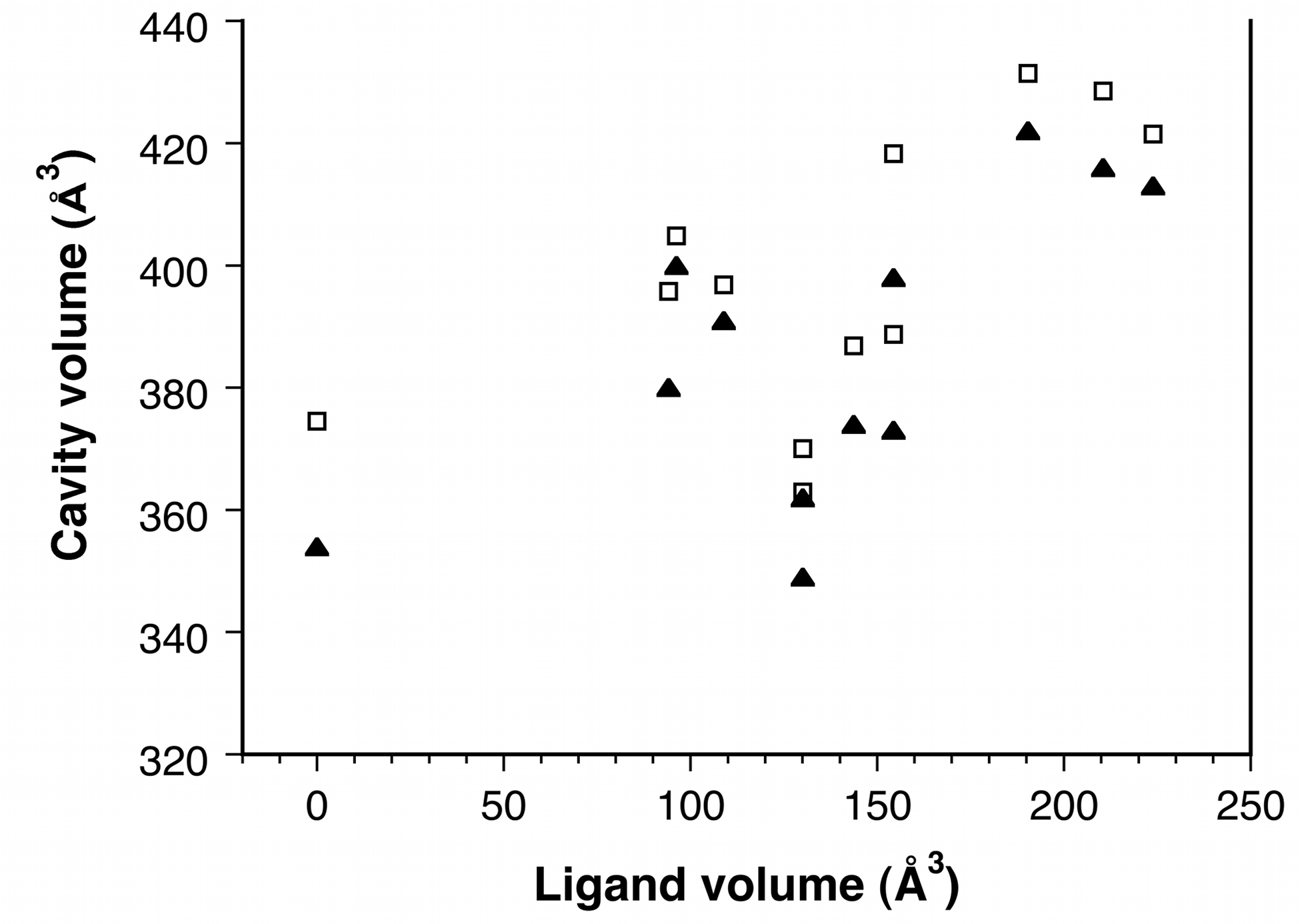

Supplement: Figure S2 — Comparison of two methods for determining apoferritin cavity volume. The crosses represent volumes calculated by choosing ten random orientations of the molecule, allowing VOIDOO to run until program termination, and averaging the volumes generated for each orientation. The squares represent volumes calculated from v(n) curves fitted to the VOIDOO output, using n = 25 (see Text S1). The overall trend is maintained for the two methods, but volumes calculated by averaging cavity volumes reported after program termination were in all cases smaller than volumes calculated by curve fitting. PDB ID codes of apoferritin-anesthetic complexes contributing to this figure: 3F32, unliganded; 1XZ1, halothane; 1XZ3, isoflurane; 3F33, propofol; 3RD0, thiopental; 3RAV, pentobarbital; 3F34 & 3F35, 2,6-diethyl phenol; 3F36, 2-isopropyl phenol; 3F37 & 3F38, 2,6-dimethyl phenol; 3F39, phenol. (TIF) [file pone.0032070.s002.tif]

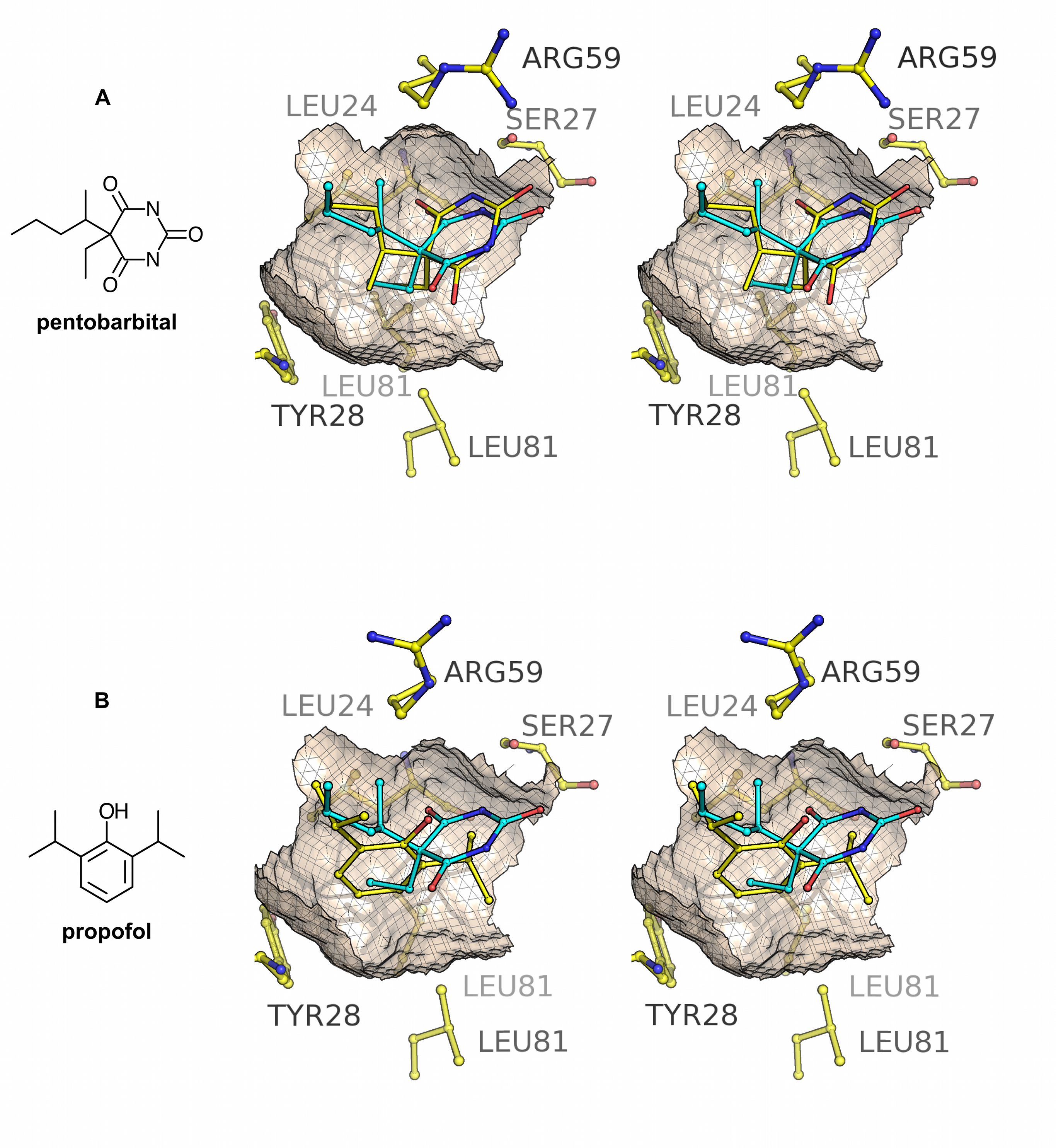

Supplement: Figure S3 — Stereo diagrams showing the hydrophobic anesthetic binding cavity of apoferritin bound to diverse general anesthetics. Pentobarbital is shown in both images, to allow direct comparison. The molecular surface of the cavity is shown as a semi-transparent tan surface that partially obscures Leu-24. A) The pentobarbital-apoferritin complex; both optical isomers are shown in this panel, in yellow and cyan. (In subsequent panels, only the more abundant isomer of pentobarbital is shown, for the sake of clarity). B) An overlay of propofol (yellow, PDB entry 3F33) and pentobarbital (cyan). (TIF) [file pone.0032070.s003.tif]

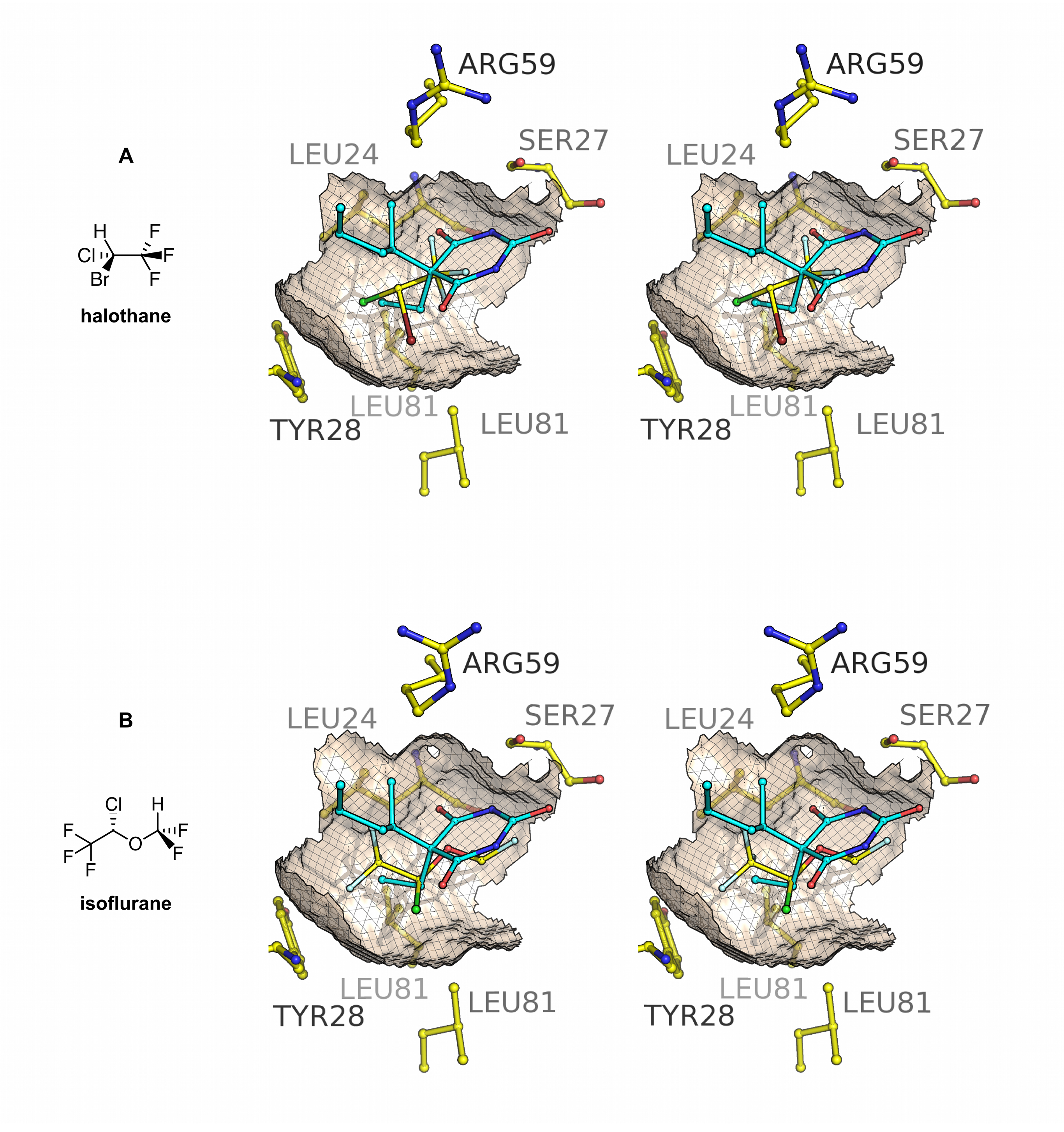

Supplement: Figure S4 — Stereo diagrams showing the hydrophobic anesthetic binding cavity of apoferritin bound to diverse general anesthetics (continued from Figure S3). Pentobarbital is included in both images, to allow direct comparison. The molecular surface of the cavity is shown as a semi-transparent tan surface that partially obscures Leu-24. A) Overlay of halothane (yellow) and pentobarbital (cyan). B) Overlay of isoflurane (yellow) and pentobarbital (cyan). (TIF) [file pone.0032070.s004.tif]

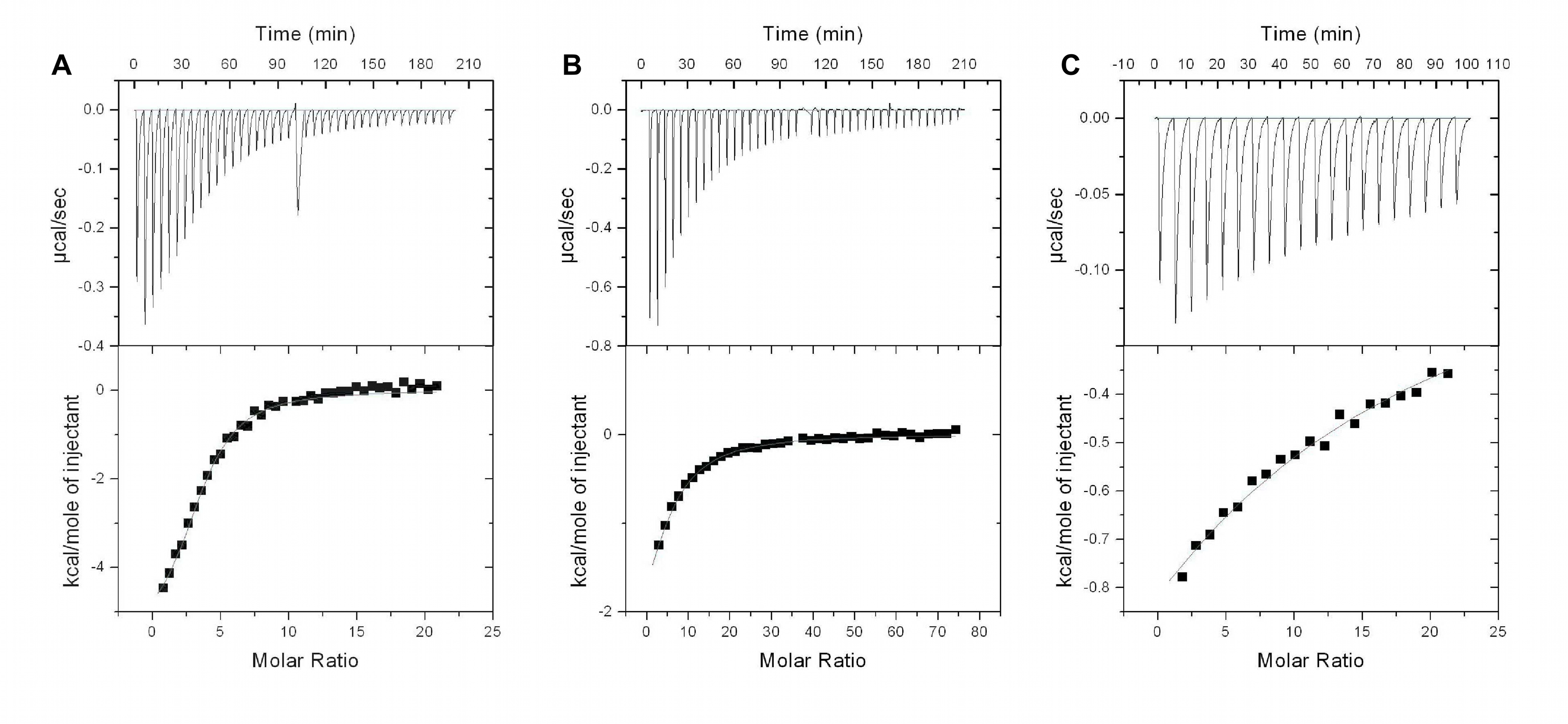

Supplement: Figure S5 — Representative ITC traces for the binding of barbiturates to apoferritin. Panel A, thiopental; panel B, pentobarbital; panel C, phenobarbital. (TIF) [file pone.0032070.s005.tif]
