# Supplementary material for: Recognition of Anesthetic Barbiturates by a Protein Binding Site: A High Resolution Structural Analysis
Source: PLoS One. 2012 Feb 16;7(2):e32070. doi: 10.1371/journal.pone.0032070 (PMC3281113; doi:10.1371/journal.pone.0032070)
Supplement: Table S1 — Apoferritin binding affinities of the optical isomers of thiopental. (DOC) [file pone.0032070.s006.doc]

***Table S1.*** Affinities of the optical isomers of thiopental for apoferritin.

| **Compound** | **Dissociation constant *K*_d_ (µM)** |
| --- | --- |
| *S*-thiopental | 18.7 ± 0.9 |
| *R*-thiopental | 29.3 ± 3.1 |
| Racemic mixture^a^ | 18.2 ± 0.2 |

*^a^* The racemic mixture in this experiment was produced by mixing equimolar amounts of purified *S*- and *R*-thiopental. Slight differences between the affinity reported here and that listed in Table 1 (which was measured for commercial (±)-thiopental) probably reflect differences between lots and/or errors in concentration measurements.
