# Supplementary material for: Recognition of Anesthetic Barbiturates by a Protein Binding Site: A High Resolution Structural Analysis
Source: PLoS One. 2012 Feb 16;7(2):e32070. doi: 10.1371/journal.pone.0032070 (PMC3281113; doi:10.1371/journal.pone.0032070)
Supplement: Text S1 — Details of cavity volume calculations, and a comparison of how different general anesthetics to the common apoferritin pharmacophore. (DOC) [file pone.0032070.s007.doc]

**Supporting Information**

Simon Oakley, Sangeetha Vedula, Weiming Bu, Qing Cheng Meng, Jin Xi, Renyu Liu, Roderic G. Eckenhoff, & Patrick J. Loll

“Recognition of Anesthetic Barbiturates by a Protein Binding Site: A High Resolution Structural Analysis”

*Cavity volume calculations.* Cavity volumes for native and ligand-bound ferritin were calculated as previously described [1]. However, while this method is more than adequate for determining the rank order of cavity volumes when comparing structures, limitations inherent within the approach complicate the estimation of absolute values of cavity volumes. Therefore, to avoid ambiguity, more details of our approach are given below.

The classic approach to estimating cavity volumes is to determine the solvent-accessible protein surface using a spherical, solvent-sized probe [2], and then determine the cavity volume enclosed by this surface. The anesthetic-binding cavity in apoferritin is linked to the solvent space by two channels; the narrowest points of these channels (bottlenecks) are located near the two symmetry-related copies of Arg-59. The diameters of these channels are close to the size of the solvent probe used for delineating the protein surface, so small variations in the positions of the atoms near the bottlenecks can make the difference between the solvent probe being trapped within the cavity or escaping from the cavity into bulk solvent. The latter case is obviously problematic for accurate calculation of cavity volume, as the boundary of the cavity is not well-defined. Therefore, dummy atoms were added to the coordinate file to ensure that the boundary between the buried cavity and the bulk solvent accessible is always clearly delineated.

To accomplish this, the cavity was filled with water molecules using FLOOD [3]. Water molecules near Arg-59 were selected to close the cavity at its narrowest point and were appended to molecular coordinates of the apoferritin dimer. To ensure uniformity, the same set of cavity-capping water molecules was used for all apoferritin-ligand complexes to define the cavity-solvent boundary.

When amino acid side chains lining the anesthetic binding site were present in two conformers, one conformation was used in one of the protomers forming the dimer, and the other conformation for the other half of the dimer.

Next, the coordinates of the apoferritin dimer structure were rotated into ten random orientations with PDBSET [4], in order to randomize the position of the initial rough grid used for cavity detection. In each orientation VOIDOO was then used to detect and refine the solvent-accessible volume of the anesthetic binding cavity. An initial grid size of 0.5 Å was used to detect the cavity; cavities were then refined by rolling a 1.4 Å radius probe over the cavity surface while the grid spacing was systematically decreased in successive cycles of the calculation [3]. Any one of three endpoint criteria would terminate VOIDOO: The calculated cavity volume changed by ≤ 0.05 Å^3^ between cycles; cavity volume changed by ≤ 0.05 % between cycles; or total number of cycles exceeded 25.

*Limitations of the cavity volume calculation, and an alternate approach.* In Cartesian space, the volume of a complex shape such as a cavity is constant, irrespective of any rotational or translational transformations. However, cavity-finding programs such as VOIDOO do not measure absolute cavity volume, but rather define cavity volume on a grid; this grid begins as a coarse sampling of the Cartesian space and becomes finer over successive program iterations. In the initial detection cycle, the detected cavity volume will vary depending upon the orientation of the molecule, because some orientations will place more initial grid points within the cavity than others. As the program cycles, the grid used to calculate cavity volume becomes finer and the detected cavity volume increases towards an asymptote, the “true” cavity volume. To average out orientation-dependent grid effects, volumes are typically calculated for many (ten or more) random molecular orientations; volumes obtained from all of these runs are then averaged.

The recommended termination criteria for VOIDOO are based on the assumption that minimal changes in detected cavity volume between refinement cycles signal approach to the asymptote. However, local plateaus can occur during the calculation, before the asymptote is reached (see arrows in Figure S1). VOIDOO will sometimes proceed past these local plateaus, but in other cases the program termination criteria are satisfied, leading to early termination. Therefore, it may be inappropriate to average data for all runs, since this gives equal weighting to runs that terminate early and runs that approach the asymptote.

This problem can be overcome by modeling the calculated cavity volume versus program cycle data using an exponential function:

(1)

where *v* = cavity volume and *n* = number of program cycles. We used gnuplot [5] to fit curves and estimate the parameters *a, b* and *k*, as shown in Figure S1. Data from the first five cycles were omitted due to wide spread. The fitted curves effectively smooth the data, providing superior estimates of the asymptote values and circumventing the problem of early program termination.

The cavity volumes presented in Table 1 of the paper approximately correspond to *v*(*n*) values obtained for *n* ≈ 15. At this point, however, the fitted curves are far from their asymptotes. At larger values of *n*, for instance *n* = 25, the values of *v*(*n*) will approach the asymptote more closely, yielding calculated cavity volumes that are on average 12.3 Å^3^ larger than the values reported in Table 1. Note that increasing the number of cycles does not alter the trend of the data (Figure S2); however, if a comparison of absolute cavity volumes between different structures is required, it will be important to choose points at lying comparable positions along their respective curves.

*Binding of different general anesthetics to the common apoferritin pharmacophore.* As shown in Figures S3 & S4, structurally different anesthetics share a common binding epitope in the apoferritin cavity, a hydrophobic patch formed by residues Leu-24, Ser-27 and Leu-81 from one monomer and Tyr-28 and Leu-81 from the other monomer.

Pentobarbital binds with its C5 carbon atom positioned in the center of the cavity; its ethyl moiety is oriented towards the hydrophobic patch, with the terminal carbon closest to Tyr-28 and the other carbon atom lying close to a groove between Leu-81 of one monomer and Leu-81 from the other monomer (panel A, Figure S3).

When the atomic coordinates of the propofol structure are overlaid with the pentobarbital structure (panel B, Figure S3), three atoms of propofol’s aromatic ring fall in analogous positions to the C5 atom and ethyl chain of pentobarbital. To a lesser extent, the hydroxyl and 1-isopropyl groups of propofol are located in similar positions to the 1-methylbutyl group of pentobarbital.

In a similar fashion, halothane’s chloride atom lies in a similar position to the terminal carbon of the ethyl moiety of pentobarbital (panel A, Figure S4). At the other end of the halothane molecule a carbon and two fluorine atoms lie in positions analogous to atoms of the barbiturate ring.

Finally, comparison of the apoferritin complexes with isoflurane and pentobarbital reveals that the trifluoromethyl group of isoflurane occupies the same position as the terminal carbon of pentobarbital’s ethyl moiety (panel A, Figure S4). The second carbon of the isoflurane molecule (which bears a chlorine substituent) overlies the other ethyl carbon of pentobarbital.

*Confirmation of space group symmetry.* In response to prompting from a reviewer, we examined whether the moderately high merging R-values in the outer shell might reflect a breakdown of the binding site’s two-fold symmetry at high resolution. Using the program XDS [6], the diffraction data for the apoferritin-thiopental complex were reintegrated and scaled in both space group *F*432 and the lower symmetry space group *F*23 (in *F*23 the two monomers that form the anesthetic binding site would not be related by crystallographic symmetry, whereas they are in *F*432).

Agreement statistics for symmetry-related reflections (as exemplified by the value *R*_merge_) were essentially identical when the data were processed in *F*23 and *F*432. In particular, the values for *R*_merge_ in the two data sets did not diverge at high resolution, indicating that the symmetry did not break down at high resolution. The overall *R*_merge_ for all data between 25 and 2.0 Å was 0.060 for the data processed as *F*23 and 0.061 for the data processed as *F*432; in the outermost (2.0—2.05 Å) shell *R*_merge_ values were 0.587 and 0.616, respectively. The minute differences probably reflect the 2-fold lower redundancy of the data processed in *F*23.

Structures of monomeric apoferritin (in space group *F*432) and dimeric apoferritin (in space group *F*23) were then refined using Phenix [7]. Input coordinates for refinement were either obtained directly from PDB entry 3RD0, or by applying the “shake” functionality in Phenix to the 3RD0 coordinates; the shake function introduced random coordinate shifts with an overall mean value of 0.3 Å into the structures before refinement. Free-R flags were not used, as refinements in the two different space groups did not utilize the same numbers of reflections. In both cases, the final results of the refinement were very similar; the lower symmetry space group showed slightly lower crystallographic R-values, as expected, since the data-to-parameter ratio is reduced by a factor of two. In *F*23, the overall R-value obtained after refinement was 0.164 for data between 25 and 2.0 Å, both with and without coordinate perturbation. In contrast, in *F*432 R-values of 0.168 and 0.174 were obtained with and without coordinate perturbation, respectively. Thus, switching to a lower symmetry space group did not drastically lower either the overall R-value. Importantly, no significant differences were seen in R-values at high resolution for the two space groups.

On the basis of these tests, we concluded that the correct space group symmetry for the apoferritin-barbiturate complex was indeed space group 209, *F*432.

**References**

1. Vedula LS, Brannigan G, Economou NJ, Xi J, Hall MA, et al. (2009) A unitary anesthetic binding site at high resolution. J Biol Chem 284: 24176-24184.

2. Connolly ML (1983) Analytical molecular surface calculation. J Appl Cryst 16: 548-558.

3. Kleywegt GJ, Jones TA (1994) Detection, delineation, measurement and display of cavities in macromolecular structures. Acta Crystallogr D Biol Crystallogr D50: 178-185.

4. Collaborative Computational Project N (1994) The CCP4 Suite: Programs for Protein Crystallography. Acta Crystallographica D50: 760-763.

5. Racine J (2006) gnuplot 4.0: A portable interactive plotting utility. Journal of Applied Econometrics 21: 133-141.

6. Kabsch W (2010) XDS. Acta Crystallogr D Biol Crystallogr 66: 125-132.

7. Adams PD, Afonine PV, Bunkoczi G, Chen VB, Davis IW, et al. (2010) PHENIX: a comprehensive Python-based system for macromolecular structure solution. Acta Crystallogr D Biol Crystallogr 66: 213-221.
